# The making of a dangerous vector: factors shaping the vector competence of *Aedes* mosquitoes

**DOI:** 10.3389/fcimb.2025.1718737

**Published:** 2025-11-04

**Authors:** Yang Wu, Jie Wu, Qipeng Wu, Huiling Cai, Jia Hu, Zhiwei Lin, Ruyan Liao

**Affiliations:** ^1^ Guangzhou Customs Technology Center, Guangzhou, China; ^2^ State Key Laboratory of Respiratory Disease, Guangzhou, China

**Keywords:** vector competence, *Aedes albopictus*, *Aedes aegypti*, chikungunya, dengue, arbovirus

## Abstract

*Aedes* mosquitoes are among the world’s most significant arbovirus vectors, transmitting pathogens such as dengue, chikungunya, and Zika viruses. However, key species like *Aedes aegypti* and *Aedes albopictus* exhibit notable differences in their vector competence, a trait of critical epidemiological importance. Vector competence is a complex phenotype, intrinsically defined by the efficiency with which a virus can overcome key tissue barriers, primarily the midgut and salivary glands. This review synthesizes the current understanding of the multifactorial drivers behind this variation through a comparative analysis of intrinsic determinants—including the vector’s genetic background, innate immunity, co-evolution with the virus, tissue barriers, and gut microbiota—and extrinsic factors, such as climatic conditions and anthropogenic pressures. By dissecting these mechanisms, this review provides a critical reference for assessing the epidemic risks of mosquito-borne diseases and aims to inform the development of more precise, next-generation vector control strategies.

## 1 Background

The global community is currently facing a formidable challenge from the spread of mosquito-borne viral diseases. The rise of emerging and re-emerging mosquito-borne diseases as a persistent global health threat is being driven by a trio of powerful global trends: accelerating globalization, climate warming, and rapid urbanization. The primary vectors in this global health crisis are *Aedes aegypti* and *Aedes albopictus*. The ongoing geographical expansion of these mosquitoes has markedly amplified the transmission risk for numerous arboviruses, including dengue (DENV), chikungunya (CHIKV), Zika (ZIKV), and yellow fever viruses (YFV). This escalating threat is evidenced by numerous large-scale epidemics. In 2014, historic dengue outbreaks were recorded across Asia, including in Japan (Quam et al., 2016), Singapore (Hapuarachchi et al., 2016), and China (Wang et al., 2016; Zhu et al., 2018), totaling over 45000 cases and severely challenging regional public health systems. More recently, the Guangdong-Hong Kong-Macao Greater Bay Area has faced immense pressure from both imported and local dengue transmission in 2024, driven by its suitable climate, dense population, and frequent commercial activities (Guangdong Provincial Center for Disease Control and Prevention, 2024). Concurrently, other *Aedes*-borne diseases have shown alarming trends. As of July 2025, a significant chikungunya fever outbreak was reported in Foshan, China, with over 3000 cases (Shunde District Health Bureau of Foshan City, 2025). Furthermore, the 2015–2016 Zika virus epidemic caused an incalculable socioeconomic and public health burden after it was confirmed to cause microcephaly and other severe neurological defects in newborns via mother-to-child transmission. This global crisis led the World Health Organization (WHO) to declare it a Public Health Emergency of International Concern (PHEIC) (Hennessey, 2016).

Vector competence is defined as the intrinsic ability of a vector to acquire a pathogen from an infected host and subsequently transmit it to a new, susceptible host (Beerntsen et al., 2000). Vector competence varies significantly among different mosquito species. For instance, *Ae. aegypti* is a highly efficient vector for arboviruses such as DENV (Kamgang et al., 2019), ZIKV (Fernandes et al., 2020), and YFV (Johnson et al., 2002), whereas *Ae. albopictus* has proven to be a more competent vector for CHIKV (Lounibos and Kramer, 2016). Furthermore, the global distributions of these two primary vectors differ substantially. *Ae. aegypti*, which originated in Africa, is now predominantly found in tropical and subtropical regions and is highly adapted to densely populated urban environments. By comparison, *Ae. albopictus* originated in Southeast Asia and has become one of the world’s most invasive species, spreading to tropical, subtropical, and temperate zones and establishing stable populations in numerous European countries (Kraemer et al., 2015). These profound differences in biology and distribution raise a critical question: what are the underlying mechanisms that determine why one mosquito species is a more effective vector than another?

## 2 The infection-dissemination-transmission pathway

**Figure 1:**
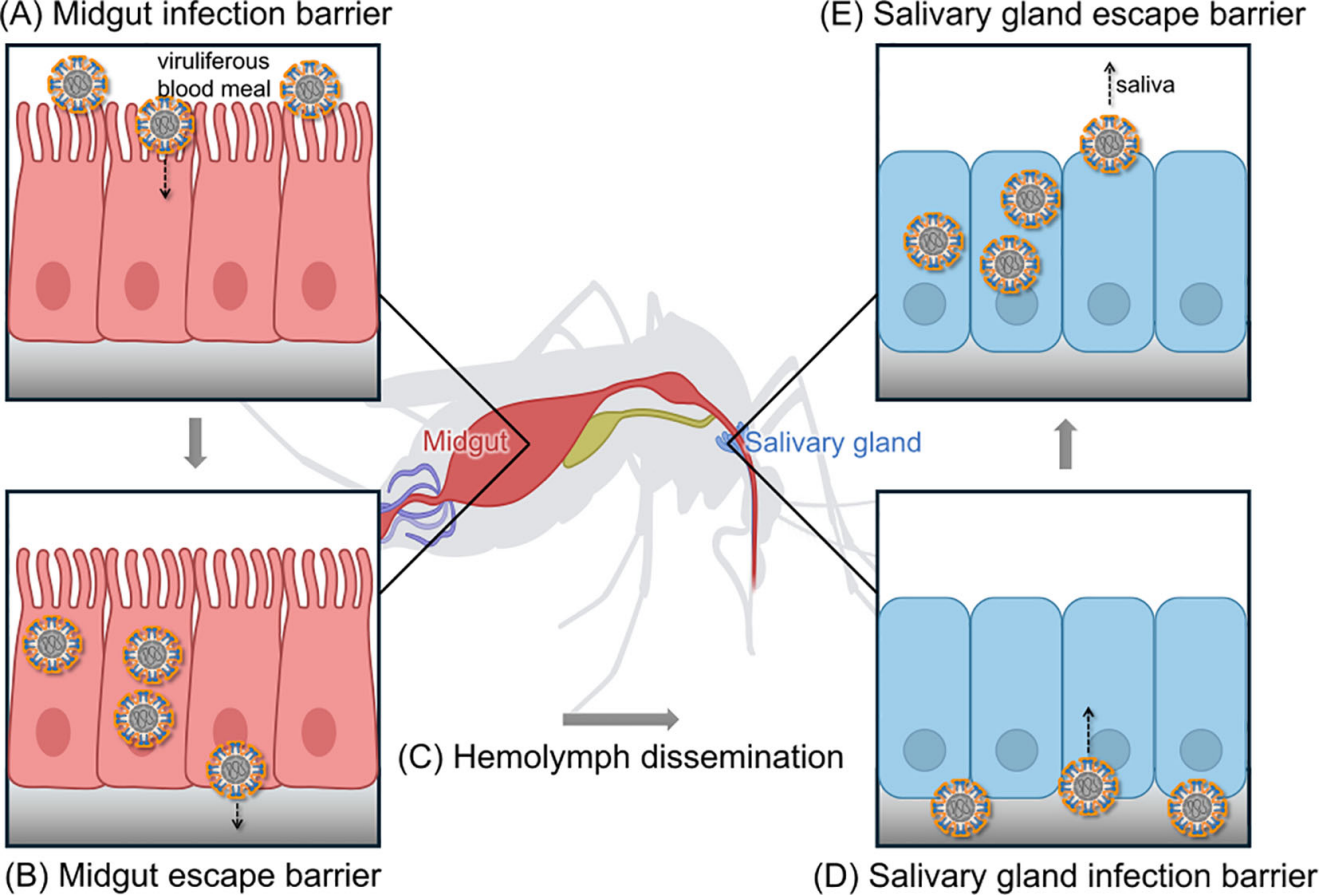
The arbovirus infection-dissemination-transmission pathway in Aedes mosquitoes. This model illustrates the key stages an arbovirus must complete to be transmitted by a mosquito vector. **(A)** Following the ingestion of a viruliferous blood meal, the virus must first infect the midgut epithelial cells, a process governed by the midgut infection barrier. **(B)** After replication, the virus must then escape the midgut and cross the basal lamina into the hemolymph, overcoming the midgut escape barrier. **(C)** The virus disseminates via the hemolymph throughout the mosquito’s body. **(D)** To become transmissible, the virus must infect the salivary glands, surmounting the salivary gland infection barrier. **(E)** Finally, the virus is released into the saliva during a subsequent blood meal, a step regulated by the salivary gland escape barrier. The efficiency of overcoming these successive barriers collectively dominates the vector competence of the mosquito.

Vector competence is a complex biological phenotype that arises from the dynamic interplay between the mosquito vector and virus. The process begins when a mosquito ingests virus by feeding on an infected host. Once inside, the pathogen’s ability to replicate and spread is tightly controlled by the mosquito vector’s own genetic background, physiology, and immune system. Ultimately, for the mosquito to become infectious, the virus must successfully navigate a series of critical tissue barriers. The efficiency of this passage is the core determinant of an individual mosquito’s vector competence (**Figure 1**).

### 2.1 Midgut infection and escape

Upon entering the mosquito midgut with a blood meal, the virus must maintain its structural integrity and infectivity within the harsh, enzyme-rich environment of the midgut lumen. The virus then recognizes and invades the midgut epithelial cells, a critical step governed by the midgut infection barrier (MIB) (Franz et al., 2015). Viral entry is dependent on binding to specific receptors on the epithelial cell surface. For instance, C-type lectins have been identified as potential cofactors for DENV attachment to the midgut epithelium in *Ae. albopictus* following a blood meal (Gao et al., 2024). Identifying and characterizing these specific host receptors is therefore a primary objective in the study of mosquito-virus interactions.

Following a successful invasion, the virus replicates within the epithelial cells. Newly synthesized viral particles are then released from the basal side of the cells and traverse the basal lamina to enter the hemolymph, the mosquito’s open circulatory system (Carpenter and Clem, 2023). This “escape” is regulated by the midgut escape barrier (MEB), the second critical hurdle for the virus (Khoo et al., 2010). The MEB is considered a primary determinant of variation in vector competence, though the precise mechanisms governing viral passage are not yet fully understood. Current hypotheses include direct penetration of the basal lamina or the use of anatomical “bypass” routes (Franz et al., 2015).

### 2.2 Hemolymph dissemination

Once the virus successfully enters the hemolymph, it disseminates rapidly throughout the mosquito’s body, infecting secondary tissues such as the fat body, neural tissue, and muscles (Cheng et al., 2022). This systemic infection is a prerequisite for the virus to reach the salivary glands. Hemocytes (mosquito blood cells) can play a dual role during *flavivirus* infections like DENV and ZIKV (Cheng et al., 2022). On one hand, they are key immune cells that combat viral infections in the hemolymph. On the other, some viruses may infect prohemocytes, using them as “Trojan horses” to facilitate systemic dissemination and replication.

### 2.3 Salivary gland infection and transmission

The final stage for the virus to become transmissible involves the successful infection of the salivary glands and subsequent release into the saliva. Viruses circulating in the hemolymph must first overcome physiological and immunological obstacles to invade the salivary gland epithelial cells, a process governed by the salivary gland infection barrier (SGIB) (Sanchez-Vargas et al., 2021). After infection, the virus replicates, and new virions are released from the apical membrane into the saliva. This release is regulated by the salivary gland escape barrier (SGEB) (Stauft et al., 2022). Critically, successful infection of the salivary glands does not guarantee transmission. Epidemiological competence is achieved only when the virus has overcome all tissue barriers and accumulates in the saliva at a titer sufficient for transmission (Guerrero et al., 2020).

## 3 Key metrics for assessing vector competence

The quantitative assessment of vector competence relies on several standardized metrics, each corresponding to a key stage in the infection-dissemination-transmission pathway (Kramer and Ciota, 2015). Under controlled laboratory conditions, these metrics convert the complex biological process into analyzable data, allowing for precise comparisons of transmission potential among different vector populations or environmental conditions. The primary metrics include the infection rate, dissemination rate, transmission rate, and extrinsic incubation period.

Infection rate refers to the proportion of mosquitoes whose midgut tissues test positive for the virus at a specific time point after ingesting an infectious blood meal. This is typically determined by exposing a cohort of mosquitoes to a blood meal with a known viral titer and subsequently testing their midgut tissues for viral nucleic acids or antigens (Chouin-Carneiro et al., 2016).

Dissemination rate is the proportion of midgut-infected mosquitoes in which the virus has successfully escaped the midgut barrier and disseminated to other parts of the body. As direct viral detection in the hemolymph is technically challenging, this rate is commonly calculated by testing distal tissues, such as the head, legs, or wings, for the presence of the virus (Chouin-Carneiro et al., 2016).

Transmission rate represents the proportion of mosquitoes capable of expectorating infectious virus in their saliva. This metric is widely considered to have the most direct epidemiological relevance. In laboratory settings, it is often determined by collecting mosquito saliva and assessing its infectivity through methods like plaque assays on susceptible cell cultures (Chouin-Carneiro et al., 2016).

Extrinsic incubation period (EIP) is the time required from when a mosquito ingests a virus-laden blood meal to when it can first transmit the infectious virus through its saliva. The duration of the EIP is a critical temporal parameter, as it determines how quickly infectious vectors appear in a population. The EIP is not a fixed biological constant; it is highly sensitive to extrinsic factors, most notably ambient temperature (Mordecai et al., 2017).

## 4 Comparative vector competence of *Ae. aegypti* and *Ae. albopictus*

Originating from Africa, *Ae. aegypti* is a highly domesticated mosquito species now established in tropical and subtropical regions worldwide (Matthews et al., 2018). It exhibits strong anthropophily and endophily, typically breeding in artificial water containers found in and around human dwellings. Its behavior of taking multiple, intermittent blood meals from humans during the day, primarily indoors, greatly enhances its efficiency as a disease vector (Facchinelli et al., 2023). Consequently, *Ae. aegypti* is considered the principal driver of urban epidemics of dengue, Zika, and yellow fever.

*Ae. albopictus*, native to Southeast Asia, presents a different ecological profile. It is one of the world’s most successful invasive species, with a geographic range extending from the tropics into temperate regions of North America and Europe (Chen et al., 2015). Unlike *Ae. aegypti*, it demonstrates greater ecological plasticity, breeding in both artificial and natural water sources like tree holes. In its feeding habits, *Ae. albopictus* is more opportunistic, feeding on a variety of vertebrate hosts in outdoor environments (Fikrig and Harrington, 2021). This generalist behavior allows it to act as a potential “bridge vector,” mediating the spillover of zoonotic viruses from animal reservoirs to human populations (Pereira-dos-Santos et al., 2020). In regions where *Ae. aegypti* is absent, such as much of China and Europe, *Ae. albopictus* is the primary vector responsible for local arbovirus outbreaks (Kraemer et al., 2015).

Both *Ae. aegypti* and *Ae. albopictus* are vectors for a wide range of viruses, primarily from the genera *Flavivirus* (family Flaviviridae), *Alphavirus* (family Togaviridae), and *Phlebovirus* (family Bunyaviridae). However, their competence for different viruses, even for different viral strains, varies significantly, which has profound epidemiological implications (**Table 1**).

**Table 1:** Comparative vector competence of Ae. aegypti and Ae. albopictus for major arboviruses.

Virus	Genus	Vector competence of *Ae. aegypti*	Vector competence of *Ae. albopictus*	Remarks
Dengue virus (DENV)	*Flavivirus*	High (primary vector)	Moderate (primary vector in some regions)	*Ae. aegypti* exhibits higher dissemination and transmission rates (Epelboin et al., 2017; Ferreira-de-Lima and Lima-Camara, 2018)
Zika virus (ZIKV)	*Flavivirus*	High (primary vector)	Moderate (primary vector in some regions)	Similar to DENV, viral titers in the saliva of *Ae. aegypti* are significantly higher than in *Ae. albopictus* (Epelboin et al., 2017; Ferreira-de-Lima and Lima-Camara, 2018).
Chikungunya virus (CHIKV)	*Alphavirus*	High (primary vector)	Very high for the E1-A226V mutant strain	The E1-A226V mutation strain is a classic example of virus-vector co-evolution (Reiter et al., 2006; Paupy et al., 2009).
Yellow fever virus (YFV)	*Flavivirus*	High (primary urban vector)	Low (potential vector)	*Ae. albopictus* has low transmission efficiency; and its role in natural transmission is unconfirmed (Amraoui et al., 2016).
Mayaro virus (MAYV)	*Alphavirus*	Moderate to high	Moderate to high	MAYV is an emerging threat in South America, where both species are potential urban vectors (Pereira et al., 2020).
Ross River virus (RRV)	*Alphavirus*	Low	Moderate	RRV is a major virus in Australia, where the invasion of *Ae. albopictus* poses a potential spillover risk (Fu et al., 2023).
Rift Valley fever virus (RVFV)	*Phlebovirus*	High	High	Both *Aedes* species are efficient primary vectors, with *Culex* mosquitoes acting as secondary vectors and other biting flies and mosquitoes as potential vectors (Brustolin et al., 2017; Lumley et al., 2017; Cêtre-Sossah et al., 2023).
West Nile virus (WNV)	*Flavivirus*	Low	Low to moderate (a primary bridge vector)	Primarily transmitted by *Culex* mosquitoes with birds as the main hosts (Vidaña et al., 2020), the role of *Ae. albopictus* as a potential bridge vector is of particular interest (Daep et al., 2014).
Japanese encephalitis virus (JEV)	*Flavivirus*	Low	Low to moderate (potential vector)	The primary vectors are *Culex* mosquitoes with pigs and migratory birds as the main hosts (Levesque et al., 2024), while *Ae. albopictus* is considered a potential secondary vector in some regions (Daep et al., 2014; Hernández-Triana et al., 2022).

### 4.1 DENV and ZIKV

For these two flaviviruses, there is a broad consensus that *Ae. aegypti* is the more efficient vector. In comparative laboratory studies, *Ae. aegypti* consistently exhibits higher dissemination and transmission rates for both DENV and ZIKV than *Ae. albopictus* (Epelboin et al., 2017; Ferreira-de-Lima and Lima-Camara, 2018).

### 4.2 CHIKV

Historically, CHIKV epidemics were primarily transmitted by *Ae. aegypti*. This dynamic shifted dramatically following the emergence of the E1-A226V mutation in the virus’s envelope protein. This single amino acid substitution greatly enhanced the virus’s adaptability to *Ae. albopictus*, making this species a highly competent, and often primary, vector in recent global outbreaks (Reiter et al., 2006; Paupy et al., 2009).

### 4.3 YFV

*Ae. aegypti* is the principal urban vector of YFV. Although *Ae. albopictus* can be infected with YFV under laboratory conditions, it is considered to have low vector competence, and its role in natural transmission cycles has not been confirmed (Amraoui et al., 2016).

### 4.4 Mayaro virus (MAYV) and Ross River virus (RRV)

MAYV and RRV are alphaviruses. For MAYV, an emerging threat in South America, both *Ae. aegypti* and *Ae. albopictus* are considered potential urban vectors (Pereira et al., 2020). For RRV, prevalent in Australia, *Ae. albopictus* displays moderate competence, while *Ae. aegypti* is a less efficient vector (Fu et al., 2023).

### 4.5 Rift Valley fever virus (RVFV)

Both *Ae. aegypti* and *Ae. albopictus* are highly competent vectors for RVFV, a phlebovirus that infects both livestock and humans. *Culex* mosquitoes act as secondary vectors, while other biting flies and mosquitoes are potential vectors (Brustolin et al., 2017; Lumley et al., 2017; Cêtre-Sossah et al., 2023).

### 4.6 West Nile virus (WNV) and Japanese encephalitis virus (JEV)

The primary vectors for WNV and JEV are *Culex* mosquitoes, which maintain transmission cycles involving birds and pigs as amplifying hosts (Vidaña et al., 2020; Levesque et al., 2024). *Ae. albopictus* may act as a secondary or bridge vector for these flaviviruses, but its competence is significantly lower than that of *Culex* species (Hernández-Triana et al., 2022). *Ae. aegypti* is generally considered a poor vector with little epidemiological significance for either virus (Daep et al., 2014).

## 5 Factors driving variation in vector competence

### 5.1 Genetic background

Vector competence is fundamentally rooted in the mosquito’s genetic background, which sets the baseline for its ability to transmit pathogens. Significant continuous variation in susceptibility to arboviruses like DENV exists both within and among different geographic populations of *Ae. aegypti* (Dickson, 2014). Instead of a simple binary outcome of being either infected or uninfected, this variation manifests as a complex spectrum, ranging from complete resistance to high susceptibility, which is characteristic of a quantitative trait. Quantitative trait locus (QTL) analysis has successfully linked this phenotypic variation to specific genomic regions in *Ae. aegypti*. These analyses revealed that the QTLs governing susceptibility to multiple viruses are clustered within five specific chromosomal regions (Severson and Behura, 2016). This clustering suggests that these genomic hotspots may contain key upstream regulatory genes or functional gene clusters of the innate immune system. These regions likely form a core genetic network that orchestrates immune responses against a range of viruses, thereby providing the fundamental molecular basis for vector competence in *Ae. aegypti*.

### 5.2 Mosquito innate immunity

Upon viral invasion, the mosquito’s innate immune system is activated to combat the infection. This defense is primarily orchestrated by four key signaling pathways: RNA interference (RNAi), Janus Kinase/Signal Transducer and Activator of Transcription (JAK/STAT), Immune Deficiency (IMD), and Toll.

The RNAi pathway, particularly through small interfering RNAs (siRNAs), represents a highly specific antiviral defense mechanism in mosquitoes. During viral replication, double-stranded RNA (dsRNA) intermediates are produced, which act as pathogen-associated molecular patterns (PAMPs). These dsRNA molecules are recognized and cleaved by the endonuclease Dicer 2 (Dcr2) into siRNAs approximately 21 bp in length (Gestuveo et al., 2022). The siRNAs are then loaded into the RNA-induced silencing complex (RISC), where they guide the Argonaute 2 (AGO2) protein to specifically degrade viral RNA that is complementary to the siRNA sequence. Through this post-transcriptional gene silencing mechanism, the RNAi pathway precisely and efficiently inhibits viral gene expression and genome replication, thereby directly limiting viral proliferation within the mosquito (Liu et al., 2019). Furthermore, *Ae. aegypti* can vertically transmit RNAi-related molecules to its offspring, establishing a form of transgenerational immunity against specific viruses (Rodriguez-Andres et al., 2024).

Alongside the specific antiviral action of RNAi, the JAK/STAT, Toll, and IMD pathways activate broad, systemic antiviral states through signal amplification cascades that lead to widespread transcriptional changes. The JAK/STAT pathway, often activated by virus-induced cytokines such as Vago, triggers the phosphorylation and nuclear translocation of STAT transcription factors. This leads to the expression of hundreds of antiviral effector genes that create a hostile intracellular environment for the virus (Yadav et al., 2023). The Toll and IMD pathways both culminate in the activation of NF-κB family transcription factors, namely Rel1 and Rel2, respectively (Tassetto et al., 2019). Activation of these pathways drives the expression of antimicrobial peptides (AMPs), including defensins and cecropins, which are secreted into the hemolymph and have demonstrated direct antiviral activities (Varjak et al., 2020).

### 5.3 Virus-vector co-evolution

The co-evolution between an arbovirus and its mosquito vector is a critical driver of epidemiological change, as genetic adaptations on either side can dramatically alter vector competence and reshape disease transmission patterns. The profound epidemiological impact of this process is best exemplified by a key mutation in CHIKV, which dramatically increased the vector competence of *Ae. albopictus* (Schuffenecker et al., 2006; Tsetsarkin et al., 2007; Vazeille et al., 2007). CHIKV comprises three major lineages: the East/Central/South African (ECSA) lineage, a West African enzootic lineage, and an Asian epidemic/endemic lineage. The highly diverse ECSA lineage gave rise to the Indian Ocean sub-lineage (IOL) (Weaver et al., 2020). A single amino acid substitution in the E1 envelope protein of the IOL, an alanine-to-valine change at position 226 (A226V), increased the virus’s transmission efficiency for *Ae. albopictus* by as much as 40-fold (Schuffenecker et al., 2006; Tsetsarkin et al., 2007; Vazeille et al., 2007). This single mutation effectively shifted the primary vector for this CHIKV lineage from *Ae. aegypti* to *Ae. albopictus*. This newly adapted virus then capitalized on the global invasive spread of *Ae. albopictus*, creating a powerful synergy that resulted in unprecedented chikungunya epidemics reaching as far as the temperate regions of Europe, including France and Italy (Tomasello and Schlagenhauf, 2013; Amraoui and Failloux, 2016; Liu et al., 2025).

To counteract the mosquito’s immune defenses, viruses have evolved sophisticated immune evasion strategies, one of the most critical being the suppression of the host’s RNAi pathway (Liu et al., 2019). DENV, for example, produces a non-coding RNA known as subgenomic *flavivirus* RNA (sfRNA). This sfRNA acts as a “molecular sponge” or competitive inhibitor, sequestering key proteins of the RNAi machinery, such as Dcr2, thereby impairing the host’s antiviral response (Zhang et al., 2019). The underlying goal of such evasion tactics is often not to completely disable the host’s immunity, but rather to achieve a modest level of suppression. This balance allows the virus to establish a persistent, non-pathogenic infection without harming the mosquito’s fitness (Samuel et al., 2023). This strategy of establishing a persistent, non-pathogenic infection allows the virus to evade immune clearance and maximize its transmission potential, perfectly illustrating the dynamic, co-evolutionary balance between the vector mosquito’s immune response and the virus’s antagonistic strategies.

### 5.4 Tissue barriers in mosquitoes

#### 5.4.1 Midgut barrier

After ingestion, the first major barrier a virus must overcome is the midgut epithelium, which it infects through a receptor-mediated process involving the binding of viral envelope proteins to specific host cell-surface receptors. Both alphaviruses and flaviviruses use C-type lectins as attachment receptors to infect mosquito midgut cells (Klimstra et al., 2003; Liu et al., 2014). C-type lectins are a family of Ca2+-dependent carbohydrate-binding proteins, such as DC-SIGN and L-SIGN.

After replicating in the epithelium, the virus must cross the basal lamina (BL) to escape the midgut. This extracellular matrix, whose pore size is smaller than a virus particle, forms the primary component of the MEB. The mechanisms by which viruses cross this barrier are generally considered under two main hypotheses: direct penetration and anatomical bypass routes.

The direct penetration hypothesis suggests that the mechanical stretching of the midgut after a large blood meal may widen gaps in the BL, allowing viruses to pass through (Kantor et al., 2018). Additionally, viral infection may induce the expression of host enzymes like matrix metalloproteinases (MMPs) and caspases, which could degrade BL components to facilitate escape (Franz et al., 2015).

The second is the “bypass” hypothesis, suggesting that viruses may use anatomical shortcuts. For instance, the mosquito tracheal system may serve as a conduit for escape, a possibility supported by the detection of DENV in the tracheal system of infected *Ae. aegypti* (Salazar et al., 2007). Another potential bypass route is the cardia, a porous region at the foregut-midgut junction that has been hypothesized to serve as a key node for viral escape (Lerdthusnee et al., 1995).

#### 5.4.2 Salivary gland barrier

The efficacy of the SGIB varies by virus. When CHIKV and ZIKV were directly injected into the thorax of *Ae. aegypti*, their salivary glands were readily infected, suggesting the SGIB is largely ineffective against these viruses. In contrast, when the same experiment was performed with DENV, a small proportion (0.5%–5.5%) of mosquitoes remained uninfected, indicating that the SGIB provides some resistance to DENV (Sanchez-Vargas et al., 2021).

The SGEB is also a critical bottleneck. Even when various strains of DENV, CHIKV, and ZIKV successfully infected the salivary glands, some mosquitoes failed to expectorate infectious virus in their saliva. This demonstrates that the SGEB can prevent even highly infectious viruses from becoming transmissible (Sanchez-Vargas et al., 2021). Specific host molecules may mediate this process. For example, salivary gland surface protein 1 (SGS1)—an abundant, 3364-amino-acid protein—is composed of a Tc toxin-like Rhs/YD shell, four receptor domains, and a set of C-terminal tandem helices, with the receptor domains thought to be critical for mediating viral escape into the saliva by facilitating virus binding (Liu et al., 2023).

### 5.5 Gut microbiota

A mosquito’s gut microbiota plays a crucial role in its physiology, influencing not only its fecundity and lifespan by providing key nutrients, but also acting as a significant regulator of its vector competence. Experiments with axenic (germ-free) *Ae. aegypti* models have revealed the importance of these microbes. Compared to their conventional counterparts, axenic mosquitoes exhibit reduced fecundity, lower metabolic rates, and extended lifespans (Harrison et al., 2023). Critically, when exposed to DENV, these germ-free mosquitoes show significantly lower midgut infection rates and viral loads, indicating a stark reduction in vector competence. This effect is potentially linked to the microbiota’s role in supplying essential nutrients, such as B vitamins, which are vital for the mosquito host (Harrison et al., 2023).

The naturally lower vector competence of *Ae. albopictus* for DENV and ZIKV compared to *Ae. aegypti* may be partly explained by differences in their native gut microbiota. For instance, a specific bacterial strain, *Enterobacter hormaechei* B17 (Eh_B17), was isolated from the gut of wild *Ae. albopictus*. This bacterium secretes a metabolite, sphingosine, that exhibits potent antiviral activity against both DENV and ZIKV. Sphingosine acts by blocking the fusion between the viral envelope and the host cell membrane, a critical early step in infection. When Eh_B17 was introduced into *Ae. aegypti*, the mosquito’s susceptibility to both viruses was significantly reduced, demonstrating that specific microbes can directly modulate vector competence (Sun et al., 2024).

The endosymbiotic bacterium *Wolbachia* is another powerful example of microbe-mediated modulation of vector competence. While widely known for its use in population suppression strategies, *Wolbachia* also significantly reduces a mosquito’s vector competence for DENV. For example, *Ae. aegypti* colonized with *Wolbachia* strains including wMel, wMelCS, and wAlbB show a strong blocking effect against DENV infection, leading to dramatically reduced vector competence, and this effect is suggested to result from multiple mechanisms, including competition for resources and priming of the mosquito’s immune system. The proposed mechanistic model suggests that *Wolbachia* may antagonistically compete with DENV for limited cellular resources essential for viral replication, while concurrently upregulating the mosquito’s innate immune pathways, thereby enabling a more rapid and effective antiviral response upon infection (Flores et al., 2020).

Taken together, on one hand, these findings highlight that gut microbes and their metabolites present novel targets and avenues for the development of novel antiviral interventions. On the other hand, they underscore the need for vigilance, as the natural co-evolution of wild mosquito populations and their microbiota could unexpectedly alter vector competence and increase the risk of disease transmission.

### 5.6 Climatic factors

Climatic factors, particularly temperature, humidity, and rainfall, dynamically influence both the geographical distribution of *Aedes* populations and their vector competence. Elevated ambient temperatures, for instance, can accelerate viral replication rates within *Ae. albopictus*, which shortens the EIP for viruses like DENV, CHIKV, and WNV, thereby enhancing vector competence and increasing transmission risk (Alto and Bettinardi, 2013). The two species also have different optimal temperature ranges for viral transmission: 21.3°C~34.0°C for *Ae. aegypti* versus a cooler 19.9°C~29.4°C for *Ae. albopictus* (Ryan et al., 2019). This thermal difference contributes to regional variations in vector competence and helps explain why the more temperate-adapted *Ae. albopictus* has become the primary vector for CHIKV in European countries such as France and Italy. Humidity is another critical factor. For example, low-humidity conditions can induce dehydration stress in *Ae. aegypti*, which in turn has been shown to increase infection and dissemination rates for ZIKV (Abu et al., 2024).

### 5.7 Anthropogenic factors

Anthropogenic factors such as urbanization, insecticide use, and industrial pollution profoundly impact the environment and can significantly alter the distribution and vector competence of *Aedes* mosquitoes. Urbanization creates abundant artificial breeding sites, such as discarded plastic containers and tires, that are ideal for *Aedes* proliferation. Concurrently, the urban heat island effect raises local temperatures, which can accelerate mosquito development, shorten the viral EIP, and ultimately enhance vector competence (Acosta, 2023).

The widespread use of chemical insecticides has imposed strong selective pressure on mosquitoes, driving the evolution of resistance through metabolic changes or target-site mutations. This resistance can be linked to vector competence, as evidenced by higher dissemination rates for both ZIKV and DENV in *Ae. aegypti* populations resistant to pyrethroid insecticides (Parker-Crockett et al., 2021). The underlying mechanism involves the pleiotropic effects of metabolic genes like cytochrome P450s, which are overexpressed in insecticide-resistant populations and whose enzymes simultaneously metabolize insecticides while also modulating key antiviral immune pathways like the Toll pathway. Furthermore, the significant metabolic resources required to maintain resistance may come at a fitness cost, diverting energy from the immune system and thus weakening the mosquito’s overall defense against viruses.

Industrial pollution is another influential anthropogenic factor, whereby exposure to heavy metals in larval breeding sites can induce physiological changes in *Aedes* mosquitoes that increase their susceptibility to viral infection as adults, thereby affecting their vector competence (Vargas et al., 2025).

## 6 Discussion

**Figure 2:**
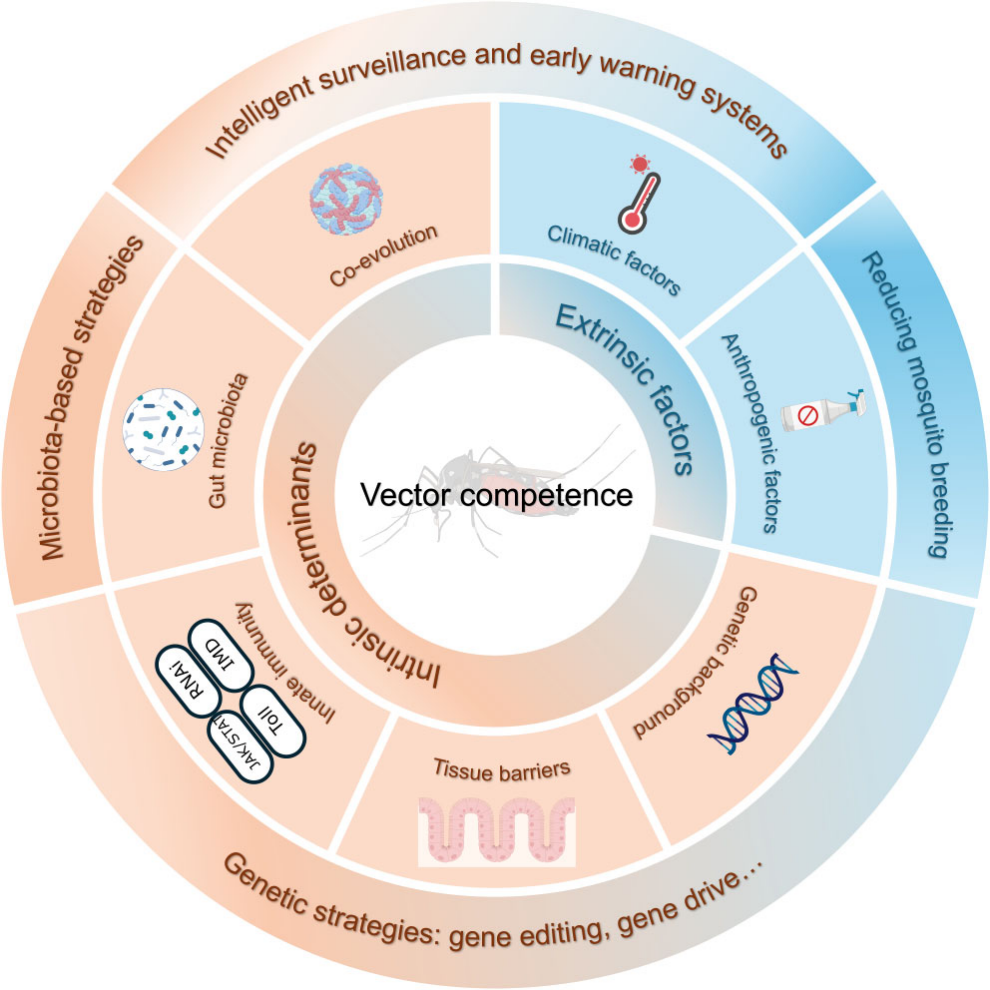
A summary of the multifactorial network shaping vector competence in *Aedes* mosquitoes. This diagram illustrates how a complex interplay of intrinsic and extrinsic factors modulates the arbovirus transmission pathway, and where different vector-control strategies can intervene. Intrinsic factors (orange) include the vector’s genetic background, innate immunity, co-evolution with the virus, tissue barriers, and gut microbiota. Extrinsic factors (blue) include climatic conditions and anthropogenic pressures. Vector-control strategies (surrounding the factors) are designed to target the transmission pathway by reducing either vector competence or mosquito breeding, which include microbiota-based and genetic strategies, intelligent surveillance, early warning systems, and conventional vector-control methods.

The vector competence of *Aedes* mosquitoes is a complex biological trait shaped by a multifactorial interplay of intrinsic and extrinsic factors, including the mosquito’s genetic background, innate immunity, and gut microbiota, as well as the specific viral strain and a range of environmental conditions (**Figure 2**). Crucially, these factors do not operate in isolation; rather, they form an intricate and dynamic regulatory network. For instance, ambient temperature directly influences the rate of viral replication, and thus the EIP, while also modulating the strength of the mosquito’s innate immune response. Similarly, the gut microbiota can alter a mosquito’s baseline immunity and susceptibility to viruses by supplying essential nutrients or secreting antiviral metabolites. Therefore, future research should adopt a systems-thinking approach that considers the dynamic interplay between the host, vector, pathogen, and environment. A crucial path forward involves integrating core biological parameters measured in the laboratory (e.g., infection and transmission rates) with key ecological parameters (e.g., vector population density, lifespan, and biting habits). Incorporating these diverse data streams into dynamic vectorial capacity models will be essential to bridge the gap from molecular mechanisms to accurate, large-scale public health risk assessments.

The significant differences in ecology and vector competence between *Ae. aegypti* and *Ae. albopictus* directly shape the global epidemiology of *Aedes*-borne viral diseases. A thorough understanding of these differences is therefore a prerequisite for conducting accurate public health risk assessments. The comparative analysis in this review highlights two distinct public health risk paradigms. In tropical regions dominated by *Ae. aegypti*, its high efficiency as a vector for viruses like DENV and ZIKV creates a high-risk paradigm for explosive, urban-centric epidemics. A different public health risk paradigm exists in the vast subtropical and temperate regions where *Ae. albopictus* is the sole vector. Here, although the baseline transmission efficiency for viruses like DENV and ZIKV is lower, a significant threat arises from the potential for viral adaptation. The emergence of the E1-A226V mutant strain of CHIKV serves as a stark warning. This single mutation transformed *Ae. albopictus* from a secondary vector into a primary driver of focal outbreaks worldwide. Given the aggressive invasive capacity of *Ae. albopictus*, this case demonstrates that even regions currently considered low-risk must remain vigilant, as viral adaptation can rapidly alter the local threat landscape. This example illustrates how the global expansion of an adaptable vector like *Ae. albopictus* provides a vast platform for viral evolution. A sudden shift in the vector competence of such a widespread species can transform a regional disease into an unpredictable global public health event, representing a major challenge for contemporary risk assessment and response strategies.

Beyond the primary global vectors, *Ae. aegypti* and *Ae. albopictus*, assessing the vector competence of other *Aedes* mosquitoes with specific traits is crucial for understanding and mitigating regional public health risks. This is particularly evident in specific ecological niches, such as the coastal wetlands of Australia, where the salt-water tolerant *Aedes vigilax* is a principal vector, exhibiting high vector competence for RRV and Barmah Forest virus (Ong et al., 2021). The role of “bridge vectors” in zoonotic spillover also highlights the critical need to evaluate their vector competence, as their feeding habits can introduce animal pathogens into human populations. For instance, the sylvatic mosquito *Aedes africanus* is a competent vector for ZIKV and YFV among primates, posing a potential transmission risk to humans at the forest edge (Oyono et al., 2022). A similar concern exists in temperate regions like Europe with the establishment of invasive species. The opportunistic feeding habits of *Aedes japonicus* on both birds and mammals, combined with its proven competence for WNV, create a significant public health threat for zoonotic transmission (Linthout et al., 2024). Furthermore, *Aedes koreicus* has been shown to be a competent vector for CHIKV and ZIKV in laboratory settings, raising concerns about its role in future outbreaks (Jansen et al., 2021). Evaluating the competence of these invasive vectors is essential not only for imported tropical pathogens but also for their potential to transmit endemic arboviruses, which could establish new local transmission cycles and reshape the regional and global arboviral disease map.

While traditional vector control strategies, which primarily aim to suppress mosquito populations, remain the cornerstone of disease control, its long-term effectiveness is increasingly challenged by the widespread evolution of insecticide resistance in mosquitoes, which diminishes the efficacy of chemical agents. Furthermore, the reliance on broad-spectrum insecticides raises ecological concerns due to their impact on non-target organisms and the broader ecosystem. These limitations highlight the necessity of integrating novel, complementary strategies, such as those aimed at reducing vector competence, to create more sustainable and resilient vector control programs. Several cutting-edge strategies are emerging, as follows:

1. Microbiota-based strategies. The most mature of these is the use of the endosymbiont *Wolbachia*. This bacterium significantly reduces the vector competence of *Aedes* for DENV and ZIKV by activating the mosquito’s immune system and competing for critical nutrients. Future work may involve genetically engineering other gut symbionts, such as *E. hormaechei*, to continuously express antiviral effector molecules. Such “population modification” approaches could serve as highly targeted vector control tools.
2. Genetic strategies. Advances in gene-editing technologies like CRISPR/Cas9, guided by a deeper understanding of the QTLs that regulate vector competence, are enabling the development of novel approaches. Gene drive systems, designed to either reduce mosquito fecundity or introduce virus-refractory traits into wild populations, offer a potential pathway to eliminate vector populations and suppress disease transmission. However, the deployment of such powerful technologies necessitates rigorous ecological risk assessments, alongside transparent community engagement and ethical oversight for responsible governance.
3. Intelligent surveillance and early warning systems. By integrating environmental data with real-time vector surveillance data, machine learning and AI models can build high-precision spatiotemporal risk prediction systems. These tools can help public health agencies allocate limited resources to high-risk areas during critical time windows, marking a shift from reactive responses to proactive forecasting.

In conclusion, a deep understanding of the mechanisms driving variation in vector competence is more than a core question for basic virology and vector biology; it serves as a critical bridge between laboratory research and public health practice. An integrated paradigm that combines molecular interventions, ecological surveillance, and intelligent risk assessment based on a holistic understanding of these mechanisms is essential. Such approaches will provide the scientific foundation needed to confront the growing global threat of *Aedes*-borne viral diseases.

## References

[B1] Abu A. E. I. Becker M. Accoti A. Sylla M. Dickson L. B. (2024). Low humidity enhances Zika virus infection and dissemination in *Aedes aegypti* mosquitoes. Msphere 9, e00401–e00424. doi: 10.1128/msphere.00401-24, PMID: 39092912 PMC11351097

[B2] Acosta E. H. (2023). Some like it hot: How urban microclimate across a tropical city impacts the capacity of Aedes mosquitoes to transmit flaviviruses. PhD Thesis, (Las Cruces: New Mexico State University).

[B3] Alto B. W. Bettinardi D. (2013). Temperature and dengue virus infection in mosquitoes: independent effects on the immature and adult stages. Am. J. Trop. Med. hygiene 88, 497. doi: 10.4269/ajtmh.12-0421, PMID: 23382163 PMC3592531

[B4] Amraoui F. Failloux A.-B. (2016). Chikungunya: an unexpected emergence in Europe. Curr. Opin. Virol. 21, 146–150. doi: 10.1016/j.coviro.2016.09.014, PMID: 27771517

[B5] Amraoui F. Vazeille M. Failloux A. B. (2016). French *Aedes albopictus* are able to transmit yellow fever virus. Eurosurveillance 21, 30361. doi: 10.2807/1560-7917.ES.2016.21.39.30361, PMID: 27719755 PMC5069433

[B6] Beerntsen B. T. James A. A. Christensen B. M. (2000). Genetics of mosquito vector competence. Microbiol. Mol. Biol. Rev. 64, 115–137. doi: 10.1128/MMBR.64.1.115-137.2000, PMID: 10704476 PMC98988

[B7] Brustolin M. Talavera S. Nuñez A. Santamaría C. Rivas R. Pujol N.. (2017). Rift Valley fever virus and European mosquitoes: vector competence of *Culex pipiens* and *Stegomyia albopicta* (= *Aedes albopictus*). Med. Veterinary Entomology 31, 365–372. doi: 10.1111/mve.12254, PMID: 28782121

[B8] Carpenter A. Clem R. J. (2023). Factors affecting arbovirus midgut escape in mosquitoes. Pathogens 12, 220. doi: 10.3390/pathogens12020220, PMID: 36839492 PMC9963182

[B9] Cêtre-Sossah C. Lebon C. Rabarison P. Cardinale E. Mavingui P. Atyame C. (2023). Evidence of Eretmapodites subsimplicipes and *Aedes albopictus* as competent vectors for Rift Valley fever virus transmission in Mayotte. Acta Tropica 239, 106835. doi: 10.1016/j.actatropica.2023.106835, PMID: 36649804

[B10] Chen X.-G. Jiang X. Gu J. Xu M. Wu Y. Deng Y.. (2015). Genome sequence of the Asian Tiger mosquito, *Aedes albopictus*, reveals insights into its biology, genetics, and evolution. Proc. Natl. Acad. Sci. 112, E5907–E5915. doi: 10.1073/pnas.1516410112, PMID: 26483478 PMC4640774

[B11] Cheng L. Liu W.-L. Su M. P. Huang S.-C. Wang J.-R. Chen C.-H. (2022). Prohemocytes are the main cells infected by dengue virus in *Aedes aegypti* and *Aedes albopictus* . Parasites Vectors 15, 137. doi: 10.1186/s13071-022-05276-w, PMID: 35449113 PMC9027048

[B12] Chouin-Carneiro T. Vega-Rua A. Vazeille M. Yebakima A. Girod R. Goindin D.. (2016). Differential susceptibilities of *Aedes aegypti* and *Aedes albopictus* from the Americas to Zika virus. PloS Negl. Trop. Dis. 10, e0004543. doi: 10.1371/journal.pntd.0004543, PMID: 26938868 PMC4777396

[B13] Daep C. A. Muñoz-Jordán J. L. Eugenin E. A. (2014). Flaviviruses, an expanding threat in public health: focus on dengue, West Nile, and Japanese encephalitis virus. J. neurovirology 20, 539–560. doi: 10.1007/s13365-014-0285-z, PMID: 25287260 PMC4331079

[B14] Dickson L. B. (2014). Population genetics and vector competence of Aedes aegypti in West Africa. PhD Thesis, (Fort Collins: Colorado State University).

[B15] Epelboin Y. Talaga S. Epelboin L. Dusfour I. (2017). Zika virus: An updated review of competent or naturally infected mosquitoes. PloS Negl. Trop. Dis. 11, e0005933. doi: 10.1371/journal.pntd.0005933, PMID: 29145400 PMC5690600

[B16] Facchinelli L. Badolo A. McCall P. J. (2023). Biology and behaviour of *Aedes aegypti* in the human environment: opportunities for vector control of arbovirus transmission. Viruses 15, 636. doi: 10.3390/v15030636, PMID: 36992346 PMC10053764

[B17] Fernandes R. S. O’Connor O. Bersot M. I. L. Girault D. Dokunengo M. R. Pocquet N.. (2020). Vector competence of *Aedes aegypti*, *Aedes albopictus* and *Culex quinquefasciatus* from Brazil and New Caledonia for three Zika virus lineages. Pathogens 9, 575. doi: 10.3390/pathogens9070575, PMID: 32708536 PMC7399907

[B18] Ferreira-de-Lima V. H. Lima-Camara T. N. (2018). Natural vertical transmission of dengue virus in *Aedes aegypti* and *Aedes albopictus*: a systematic review. Parasites Vectors 11, 77. doi: 10.1186/s13071-018-2643-9, PMID: 29391071 PMC5793400

[B19] Fikrig K. Harrington L. C. (2021). Understanding and interpreting mosquito blood feeding studies: the case of *Aedes albopictus* . Trends Parasitol. 37, 959–975. doi: 10.1016/j.pt.2021.07.013, PMID: 34497032

[B20] Flores H. A. Taneja de Bruyne J. O’Donnell T. B. Tuyet Nhu V. Thi Giang N. Thi Xuan Trang H.. (2020). Multiple *Wolbachia* strains provide comparative levels of protection against dengue virus infection in *Aedes aegypti* . PloS Pathog. 16, e1008433. doi: 10.1371/journal.ppat.1008433, PMID: 32282862 PMC7179939

[B21] Franz A. W. Kantor A. M. Passarelli A. L. Clem R. J. (2015). Tissue barriers to arbovirus infection in mosquitoes. Viruses 7, 3741–3767. doi: 10.3390/v7072795, PMID: 26184281 PMC4517124

[B22] Fu J. Y. L. Chua C. L. Abu Bakar A. S. Vythilingam I. Wan Sulaiman W. Y. Alphey L.. (2023). Susceptibility of *Aedes albopictus*, *Ae. aegypti* and human populations to Ross River virus in Kuala Lumpur, Malaysia. PloS Negl. Trop. Dis. 17, e0011423. doi: 10.1371/journal.pntd.0011423, PMID: 37307291 PMC10289418

[B23] Gao S. Xu H. Li H. Feng X. Zhou J. Guo R.. (2024). Identification and functional analysis of C-type lectin from mosquito *Aedes albopictus* in response to dengue virus infection. Parasites Vectors 17, 375. doi: 10.1186/s13071-024-06453-9, PMID: 39232769 PMC11373435

[B24] Gestuveo R. J. Parry R. Dickson L. B. Lequime S. Sreenu V. B. Arnold M. J.. (2022). Mutational analysis of *Aedes aegypti* Dicer 2 provides insights into the biogenesis of antiviral exogenous small interfering RNAs. PloS Pathog. 18, e1010202. doi: 10.1371/journal.ppat.1010202, PMID: 34990484 PMC8769306

[B25] Guangdong Provincial Center for Disease Control and Prevention (2024). Dengue Fever Surveillance Update for Guangdong Province (Week 40). Available online at: https://cdcp.gd.gov.cn/zwgk/sjfb/content/post_4504741.html (Accessed July 25, 2025).

[B26] Guerrero D. Cantaert T. Missé D. (2020). *Aedes* mosquito salivary components and their effect on the immune response to arboviruses. Front. Cell. infection Microbiol. 10, 407. doi: 10.3389/fcimb.2020.00407, PMID: 32850501 PMC7426362

[B27] Hapuarachchi H. C. Koo C. Rajarethinam J. Chong C.-S. Lin C. Yap G.. (2016). Epidemic resurgence of dengue fever in Singapore in 2013-2014: A virological and entomological perspective. BMC Infect. Dis. 16, 300. doi: 10.1186/s12879-016-1606-z, PMID: 27316694 PMC4912763

[B28] Harrison R. E. Yang X. Eum J. H. Martinson V. G. Dou X. Valzania L.. (2023). The mosquito *Aedes aegypti* requires a gut microbiota for normal fecundity, longevity and vector competence. Commun. Biol. 6, 1154. doi: 10.1038/s42003-023-05545-z, PMID: 37957247 PMC10643675

[B29] Hennessey M. (2016). Zika virus spreads to new areas—region of the Americas, May 2015–January 2016. MMWR. Morbidity mortality weekly Rep. 65, 55–58. doi: 10.15585/mmwr.mm6503e1, PMID: 26820163

[B30] Hernández-Triana L. M. Folly A. J. Sewgobind S. Lean F. Z. Ackroyd S. Nuñez A.. (2022). Susceptibility of *Aedes albopictus* and *Culex quinquefasciatus* to Japanese encephalitis virus. Parasites Vectors 15, 210. doi: 10.1186/s13071-022-05329-0, PMID: 35710580 PMC9204976

[B31] Jansen S. Cadar D. Lühken R. Pfitzner W. P. Jöst H. Oerther S.. (2021). Vector competence of the invasive mosquito species *Aedes koreicus* for arboviruses and interference with a novel insect specific virus. Viruses 13, 2507. doi: 10.3390/v13122507, PMID: 34960776 PMC8704790

[B32] Johnson B. W. Chambers T. V. Crabtree M. B. Filippis A. M. Vilarinhos P. T. Resende M. C.. (2002). Vector competence of Brazilian *Aedes aegypti* and *Ae. albopictus* for a Brazilian yellow fever virus isolate. Trans. R. Soc. Trop. Med. hygiene 96, 611–613. doi: 10.1016/S0035-9203(02)90326-3, PMID: 12625133

[B33] Kamgang B. Vazeille M. Tedjou A. N. Wilson-Bahun T. A. Yougang A. P. Mousson L.. (2019). Risk of dengue in Central Africa: Vector competence studies with *Aedes aegypti* and *Aedes albopictus* (Diptera: Culicidae) populations and dengue 2 virus. PloS Negl. Trop. Dis. 13, e0007985. doi: 10.1371/journal.pntd.0007985, PMID: 31887138 PMC6953884

[B34] Kantor A. M. Grant D. G. Balaraman V. White T. A. Franz A. W. (2018). Ultrastructural analysis of chikungunya virus dissemination from the midgut of the yellow fever mosquito, *Aedes aegypti* . Viruses 10, 571. doi: 10.3390/v10100571, PMID: 30340365 PMC6213114

[B35] Khoo C. C. Piper J. Sanchez-Vargas I. Olson K. E. Franz A. W. (2010). The RNA interference pathway affects midgut infection-and escape barriers for Sindbis virus in *Aedes aegypti* . BMC Microbiol. 10, 130. doi: 10.1186/1471-2180-10-130, PMID: 20426860 PMC2877022

[B36] Klimstra W. B. Nangle E. M. Smith M. S. Yurochko A. D. Ryman K. D. (2003). DC-SIGN and L-SIGN can act as attachment receptors for alphaviruses and distinguish between mosquito cell-and mammalian cell-derived viruses. J. Virol. 77, 12022–12032. doi: 10.1128/JVI.77.22.12022-12032.2003, PMID: 14581539 PMC254289

[B37] Kraemer M. U. Sinka M. E. Duda K. A. Mylne A. Q. Shearer F. M. Barker C. M.. (2015). The global distribution of the arbovirus vectors *Aedes aegypti* and *Ae. albopictus* . elife 4, e08347. doi: 10.7554/eLife.08347, PMID: 26126267 PMC4493616

[B38] Kramer L. D. Ciota A. T. (2015). Dissecting vectorial capacity for mosquito-borne viruses. Curr. Opin. Virol. 15, 112–118. doi: 10.1016/j.coviro.2015.10.003, PMID: 26569343 PMC4688158

[B39] Lerdthusnee K. Romoser W. S. Faran M. E. Dohm D. J. (1995). Rift Valley fever virus in the cardia of *Culex pipiens*: an immunocytochemical and ultrastructural study. Am. J. Trop. Med. hygiene 53, 331–337. doi: 10.4269/ajtmh.1995.53.331, PMID: 7485683

[B40] Levesque Z. A. Walsh M. G. Webb C. E. Zadoks R. N. Brookes V. J. (2024). A scoping review of evidence of naturally occurring Japanese encephalitis infection in vertebrate animals other than humans, ardeid birds and pigs. PloS Negl. Trop. Dis. 18, e0012510. doi: 10.1371/journal.pntd.0012510, PMID: 39365832 PMC11482687

[B41] Linthout C. Martins A. D. de Wit M. Delecroix C. Abbo S. R. Pijlman G. P.. (2024). The potential role of the Asian bush mosquito *Aedes japonicus* as spillover vector for West Nile virus in the Netherlands. Parasites Vectors 17, 262. doi: 10.1186/s13071-024-06279-5, PMID: 38886805 PMC11181672

[B42] Liu Q. Shen H. Gu L. Yuan H. Zhu W. (2025). Chikungunya virus in Europe: A retrospective epidemiology study from 2007 to 2023. PloS Negl. Trop. Dis. 19, e0012904. doi: 10.1371/journal.pntd.0012904, PMID: 40053531 PMC11906167

[B43] Liu J. Swevers L. Kolliopoulou A. Smagghe G. (2019). Arboviruses and the challenge to establish systemic and persistent infections in competent mosquito vectors: the interaction with the RNAi mechanism. Front. Physiol. 10, 890. doi: 10.3389/fphys.2019.00890, PMID: 31354527 PMC6638189

[B44] Liu S. Xia X. Calvo E. Zhou Z. H. (2023). Native structure of mosquito salivary protein uncovers domains relevant to pathogen transmission. Nat. Commun. 14, 899. doi: 10.1038/s41467-023-36577-y, PMID: 36797290 PMC9935623

[B45] Liu Y. Zhang F. Liu J. Xiao X. Zhang S. Qin C.. (2014). Transmission-blocking antibodies against mosquito C-type lectins for dengue prevention. PloS Pathog. 10, e1003931. doi: 10.1371/journal.ppat.1003931, PMID: 24550728 PMC3923773

[B46] Lounibos L. P. Kramer L. D. (2016). Invasiveness of *Aedes aegypti* and *Aedes albopictus* and vectorial capacity for chikungunya virus. J. Infect. Dis. 214, S453–S458. doi: 10.1093/infdis/jiw285, PMID: 27920173 PMC5137242

[B47] Lumley S. Horton D. L. Hernandez-Triana L. L. M. Johnson N. Fooks A. R. Hewson R. (2017). Rift Valley fever virus: strategies for maintenance, survival and vertical transmission in mosquitoes. J. Gen. Virol. 98, 875–887. doi: 10.1099/jgv.0.000765, PMID: 28555542

[B48] Matthews B. J. Dudchenko O. Kingan S. B. Koren S. Antoshechkin I. Crawford J. E.. (2018). Improved reference genome of *Aedes aegypti* informs arbovirus vector control. Nature 563, 501–507. doi: 10.1038/s41586-018-0692-z, PMID: 30429615 PMC6421076

[B49] Mordecai E. A. Cohen J. M. Evans M. V. Gudapati P. Johnson L. R. Lippi C. A.. (2017). Detecting the impact of temperature on transmission of Zika, dengue, and chikungunya using mechanistic models. PloS Negl. Trop. Dis. 11, e0005568. doi: 10.1371/journal.pntd.0005568, PMID: 28448507 PMC5423694

[B50] Ong O. T. Skinner E. B. Johnson B. J. Old J. M. (2021). Mosquito-borne viruses and non-human vertebrates in Australia: A review. Viruses 13, 265. doi: 10.3390/v13020265, PMID: 33572234 PMC7915788

[B51] Oyono M. G. Kenmoe S. Abanda N. N. Takuissu G. R. Ebogo-Belobo J. T. Kenfack-Momo R.. (2022). Epidemiology of yellow fever virus in humans, arthropods, and non-human primates in sub-Saharan Africa: A systematic review and meta-analysis. PloS Negl. Trop. Dis. 16, e0010610. doi: 10.1371/journal.pntd.0010610, PMID: 35867659 PMC9307179

[B52] Parker-Crockett C. Connelly C. R. Siegfried B. Alto B. (2021). Influence of pyrethroid resistance on vector competency for Zika virus by *Aedes aegypti* (Diptera: Culicidae). J. Med. entomology 58, 1908–1916. doi: 10.1093/jme/tjab035, PMID: 33724374

[B53] Paupy C. Delatte H. Bagny L. Corbel V. Fontenille D. (2009). *Aedes albopictus*, an arbovirus vector: from the darkness to the light. Microbes infection 11, 1177–1185. doi: 10.1016/j.micinf.2009.05.005, PMID: 19450706

[B54] Pereira T. N. Carvalho F. D. De Mendonça S. F. Rocha M. N. Moreira L. A. (2020). Vector competence of *Aedes aegypti*, *Aedes albopictus*, and *Culex quinquefasciatus* mosquitoes for Mayaro virus. PloS Negl. Trop. Dis. 14, e0007518. doi: 10.1371/journal.pntd.0007518, PMID: 32287269 PMC7182273

[B55] Pereira-dos-Santos T. Roiz D. Lourenço-de-Oliveira R. Paupy C. (2020). A systematic review: is *Aedes albopictus* an efficient bridge vector for zoonotic arboviruses? Pathogens 9, 266. doi: 10.3390/pathogens9040266, PMID: 32272651 PMC7238240

[B56] Quam M. B. Sessions O. Kamaraj U. S. Rocklöv J. Wilder-Smith A. (2016). Dissecting Japan’s dengue outbreak in 2014. Am. J. Trop. Med. hygiene 94, 409. doi: 10.4269/ajtmh.15-0468, PMID: 26711518 PMC4751952

[B57] Reiter P. Fontenille D. Paupy C. (2006). *Aedes albopictus* as an epidemic vector of chikungunya virus: another emerging problem? Lancet Infect. Dis. 6, 463–464. doi: 10.1016/S1473-3099(06)70531-X, PMID: 16870524

[B58] Rodriguez-Andres J. Axford J. Hoffmann A. Fazakerley J. (2024). Mosquito transgenerational antiviral immunity is mediated by vertical transfer of virus DNA sequences and RNAi. Iscience 27, 108598. doi: 10.1016/j.isci.2023.108598, PMID: 38155780 PMC10753076

[B59] Ryan S. J. Carlson C. J. Mordecai E. A. Johnson L. R. (2019). Global expansion and redistribution of *Aedes*-borne virus transmission risk with climate change. PloS Negl. Trop. Dis. 13, e0007213. doi: 10.1371/journal.pntd.0007213, PMID: 30921321 PMC6438455

[B60] Salazar M. I. Richardson J. H. Sánchez-Vargas I. Olson K. E. Beaty B. J. (2007). Dengue virus type 2: replication and tropisms in orally infected *Aedes aegypti* mosquitoes. BMC Microbiol. 7, 9. doi: 10.1186/1471-2180-7-9, PMID: 17263893 PMC1797809

[B61] Samuel G. H. Pohlenz T. Dong Y. Coskun N. Adelman Z. N. Dimopoulos G.. (2023). RNA interference is essential to modulating the pathogenesis of mosquito-borne viruses in the yellow fever mosquito *Aedes aegypti* . Proc. Natl. Acad. Sci. 120, e2213701120. doi: 10.1073/pnas.2213701120, PMID: 36893279 PMC10089172

[B62] Sanchez-Vargas I. Olson K. E. Black W. C. IV (2021). The genetic basis for salivary gland barriers to arboviral transmission. Insects 12, 73. doi: 10.3390/insects12010073, PMID: 33467430 PMC7830681

[B63] Schuffenecker I. Iteman I. Michault A. Murri S. Frangeul L. Vaney M.-C.. (2006). Genome microevolution of chikungunya viruses causing the Indian Ocean outbreak. PloS Med. 3, e263. doi: 10.1371/journal.pmed.0030263, PMID: 16700631 PMC1463904

[B64] Severson D. W. Behura S. K. (2016). Genome investigations of vector competence in *Aedes aegypti* to inform novel arbovirus disease control approaches. Insects 7, 58. doi: 10.3390/insects7040058, PMID: 27809220 PMC5198206

[B65] Shunde District Health Bureau of Foshan City (2025). Official Statement. Available online at: https://cdcp.gd.gov.cn/ywdt/zdzt/yfjkkyr/yqxx/content/post_4749264.html (Accessed July 25, 2025).

[B66] Stauft C. B. Phillips A. T. Wang T. T. Olson K. E. (2022). Identification of salivary gland escape barriers to western equine encephalitis virus in the natural vector, *Culex tarsalis* . PloS One 17, e0262967. doi: 10.1371/journal.pone.0262967, PMID: 35298486 PMC8929657

[B67] Sun X. Wang Y. Yuan F. Zhang Y. Kang X. Sun J.. (2024). Gut symbiont-derived sphingosine modulates vector competence in *Aedes* mosquitoes. Nat. Commun. 15, 8221. doi: 10.1038/s41467-024-52566-1, PMID: 39300135 PMC11413220

[B68] Tassetto M. Kunitomi M. Whitfield Z. J. Dolan P. T. Sánchez-Vargas I. Garcia-Knight M.. (2019). Control of RNA viruses in mosquito cells through the acquisition of vDNA and endogenous viral elements. elife 8, e41244. doi: 10.7554/eLife.41244.026, PMID: 31621580 PMC6797480

[B69] Tomasello D. Schlagenhauf P. (2013). Chikungunya and dengue autochthonous cases in Europe 2007–2012. Travel Med. Infect. Dis. 11, 274–284. doi: 10.1016/j.tmaid.2013.07.006, PMID: 23962447

[B70] Tsetsarkin K. A. Vanlandingham D. L. McGee C. E. Higgs S. (2007). A single mutation in chikungunya virus affects vector specificity and epidemic potential. PloS Pathog. 3, e201. doi: 10.1371/journal.ppat.0030201, PMID: 18069894 PMC2134949

[B71] Vargas V. García-Martínez R. Nava-Castro K. E. Garay-Canales C. A. Cime-Castillo J. Lanz-Mendoza H.. (2025). Detection of heavy metals in various stages of development for wild mosquitoes of *Aedes aegypti* and *Aedes albopictus* sourced from artificial aquatic niches in arbovirus endemic areas. Sci. total Environ. 981, 179551. doi: 10.1016/j.scitotenv.2025.179551, PMID: 40347752

[B72] Varjak M. Gestuveo R. J. Burchmore R. Schnettler E. Kohl A. (2020). aBravo is a novel *Aedes aegypti* antiviral protein that interacts with, but acts independently of, the exogenous siRNA pathway effector dicer 2. Viruses 12, 748. doi: 10.3390/v12070748, PMID: 32664591 PMC7411624

[B73] Vazeille M. Moutailler S. Coudrier D. Rousseaux C. Khun H. Huerre M.. (2007). Two Chikungunya isolates from the outbreak of La Reunion (Indian Ocean) exhibit different patterns of infection in the mosquito, *Aedes albopictus* . PloS One 2, e1168. doi: 10.1371/journal.pone.0001168, PMID: 18000540 PMC2064959

[B74] Vidaña B. Busquets N. Napp S. Pérez-Ramírez E. Jiménez-Clavero M.Á. Johnson N. (2020). The role of birds of prey in West Nile virus epidemiology. Vaccines 8, 550. doi: 10.3390/vaccines8030550, PMID: 32967268 PMC7564710

[B75] Wang S.-F. Chang K. Loh E.-W. Wang W.-H. Tseng S.-P. Lu P.-L.. (2016). Consecutive large dengue outbreaks in Taiwan in 2014–2015. Emerging Microbes infections 5, 1–3. doi: 10.1038/emi.2016.124, PMID: 27924810 PMC5180368

[B76] Weaver S. C. Chen R. Diallo M. (2020). Chikungunya virus: role of vectors in emergence from enzootic cycles. Annu. Rev. entomology 65, 313–332. doi: 10.1146/annurev-ento-011019-025207, PMID: 31594410

[B77] Yadav M. Dahiya N. Sehrawat N. (2023). Mosquito gene targeted RNAi studies for vector control. Funct. Integr. Genomics 23, 180. doi: 10.1007/s10142-023-01072-6, PMID: 37227504 PMC10211311

[B78] Zhang Y. Zhang Y. Liu Z. Y. Cheng M. L. Ma J. Wang Y.. (2019). Long non-coding subgenomic flavivirus RNAs have extended 3D structures and are flexible in solution. EMBO Rep. 20, e47016. doi: 10.15252/embr.201847016, PMID: 31502753 PMC6832101

[B79] Zhu G. Xiao J. Zhang B. Liu T. Lin H. Li X.. (2018). The spatiotemporal transmission of dengue and its driving mechanism: A case study on the 2014 dengue outbreak in Guangdong, China. Sci. total Environ. 622, 252–259. doi: 10.1016/j.scitotenv.2017.11.314, PMID: 29216466

